# Temporal Genetic Variance and Propagule-Driven Genetic Structure Characterize Naturalized Rainbow Trout (*Oncorhynchus mykiss*) from a Patagonian Lake Impacted by Trout Farming

**DOI:** 10.1371/journal.pone.0142040

**Published:** 2015-11-06

**Authors:** Javiera N. Benavente, Lisa W. Seeb, James E. Seeb, Ivan Arismendi, Cristián E. Hernández, Gonzalo Gajardo, Ricardo Galleguillos, Maria I. Cádiz, Selim S. Musleh, Daniel Gomez-Uchida

**Affiliations:** 1 Department of Zoology, Universidad de Concepcion, Casilla 160-C, Concepcion, Chile; 2 School of Aquatic and Fishery Sciences, University of Washington, Box 355020, Seattle, WA, 98195-5020, United States of America; 3 Department of Fisheries & Wildlife, Oregon State University, 104 Nash Hall, 2820 SW Campus Way, Corvallis, OR, 97331, United States of America; 4 Laboratorio de Genética, Acuicultura & Biodiversidad, Universidad de Los Lagos, Osorno, Chile; 5 Department of Oceanography, Universidad de Concepcion, Casilla 160-C, Concepcion, Chile; 6 Interdisciplinary Center for Aquaculture Research (INCAR), Barrio Universitario s/n, Universidad de Concepcion, Concepcion, Chile; SOUTHWEST UNIVERSITY, CHINA

## Abstract

Knowledge about the genetic underpinnings of invasions—a theme addressed by invasion genetics as a discipline—is still scarce amid well documented ecological impacts of non-native species on ecosystems of Patagonia in South America. One of the most invasive species in Patagonia’s freshwater systems and elsewhere is rainbow trout (*Oncorhynchus mykiss*). This species was introduced to Chile during the early twentieth century for stocking and promoting recreational fishing; during the late twentieth century was reintroduced for farming purposes and is now naturalized. We used population- and individual-based inference from single nucleotide polymorphisms (SNPs) to illuminate three objectives related to the establishment and naturalization of Rainbow Trout in Lake Llanquihue. This lake has been intensively used for trout farming during the last three decades. Our results emanate from samples collected from five inlet streams over two seasons, winter and spring. First, we found that significant intra- population (temporal) genetic variance was greater than inter-population (spatial) genetic variance, downplaying the importance of spatial divergence during the process of naturalization. Allele frequency differences between cohorts, consistent with variation in fish length between spring and winter collections, might explain temporal genetic differences. Second, individual-based Bayesian clustering suggested that genetic structure within Lake Llanquihue was largely driven by putative farm propagules found at one single stream during spring, but not in winter. This suggests that farm broodstock might migrate upstream to breed during spring at that particular stream. It is unclear whether interbreeding has occurred between “pure” naturalized and farm trout in this and other streams. Third, estimates of the annual number of breeders (*N*_*b*_) were below 73 in half of the collections, suggestive of genetically small and recently founded populations that might experience substantial genetic drift. Our results reinforce the notion that naturalized trout originated recently from a small yet genetically diverse source and that farm propagules might have played a significant role in the invasion of Rainbow Trout within a single lake with intensive trout farming. Our results also argue for proficient mitigation measures that include management of escapes and strategies to minimize unintentional releases from farm facilities.

## Introduction

Releases of non-native species can cause economic losses and ecological impacts to native ecosystems [1–3]. However, propagation of non-natives are likely to continue if these activities are considered beneficial or profitable [4]. Species that become invasive can be harmful to freshwater ecosystems [5,6], and the case of salmonids (i.e., salmon and trout) introduced to Patagonia in South America is especially insightful [7,8]. Revenue from recreational fishing of salmonids in both Chile and Argentina—the only two countries where salmonids have hitherto established—have been appraised in millions of dollars annually [9]. This prompted multiple publicly and privately funded initiatives to stock Patagonian rivers and lakes [10–13]. Additionally, profits from farming of salmonids in South America are even higher as Chile, in particular, has become the world’s second largest producer of farmed salmonids with revenue in the order of billions of dollars [9,14,15]. Farming is another important source of propagules mediating the establishment and naturalization of salmonids in this region as certain methods of cultivation and husbandry of farmed fish are often linked to invasions [2,16,17].

Knowledge about the genetic underpinnings of the invasion of salmonids in Patagonia has been recently accumulating [17–20] amid many studies addressing ecological effects of salmonids on native fishes, including predation and competition [21–27]. Invasion genetics, the investigation of genetic variation among non-native populations and consequences for their ecology and evolution in novel environments, is still considered an emerging discipline [28]. Investigation of genetic variation using various molecular methods can be used to: (i) evaluate founding events on introduced populations [29], (ii) gauge the consequences of population admixture on neutral genetic diversity [17] and on individual fitness [30], or (iii) trace the history of human-mediated introductions back to their donor populations [31–33]. Non-equilibrium and individual-based approaches have provided powerful insights into elucidating spatial genetic structure and hybridization among invasive populations [17,34–36], and such approaches might be more suitable for recently founded populations [35,37]. Parameters of population stochasticity, namely effective population size and the annual number of breeders (*N*_*b*_: [38]), are also crucial to predict the intensity of genetic drift or whether populations are capable of persisting in the face of environmental change [39], but estimation of these parameters has been largely absent from invasion genetics studies (but see [17] for an exception).

Rainbow Trout (*Oncorhynchus mykiss*) is one of the most notorious invasive species around the world [40] and possibly the most conspicuous salmonid in South America based on estimates of abundance [8,22]. Successful colonization of Rainbow Trout in Patagonia was likely the result of continuous propagule pressure, high phenotypic plasticity, and low environmental resistance [41]. Two stages can be identified in the introduction and naturalization of Rainbow Trout in the case of Chile: stocking of rivers in the early twentieth century, followed by new introductions through releases from farms (intentional or unintentional) during the late twentieth century [42].

Lake Llanquihue in the Lake District (X Region) is an exemplary case to illustrate these stages (Table 1). Following a stocking phase, Lake Llanquihue became Chile’s largest smolt producer of a domestic broodstock of Rainbow Trout [15]. Naturalized Rainbow Trout in Lake Llanquihue showed a resident but migratory (ad-fluvial) life history; mature adults used streams to breed during the austral winter and early spring, whereas juveniles used streams as nursery areas for one or two years before emigrating to the lake to continue feeding [42]. Reproductive isolation among newly established salmon populations might evolve in tens of generations [43]. We investigate whether genetic divergence has arisen among Rainbow Trout populating inlet streams of Lake Llanquihue and whether spatial divergence is temporally stable, often a trademark among native salmon and trout populations with large *N*_*b*_ [44,45].

**Table 1:** Brief history of Rainbow Trout (*O*. *mykiss*) introductions in Lake Llanquihue, Lake District (X Region) in Chile. Modified from [13] and [15].

Period	Description	Remarks
1910–1916	“Temporary” hatchery located at the outlet of the lake (River Maullín) that maintained 50,000–100,000 eggs imported from Germany	For stocking of River Maullín
1969–1972	First farm in the south shore that bred 37,500 adults in ten net pens for the domestic market	“Massive” escapes reported
1975–1979	Second farm located near River Pescado^a^, one inlet stream	Exported 40,000 kg of adults for the French market
1980–2014	Explosive growth of the farming industry; 15 farming companies currently authorized to maintain and breed trout	500,000 smolts produced annually for the domestic market

^a^ One of the streams in our study.

**Fig 1:**
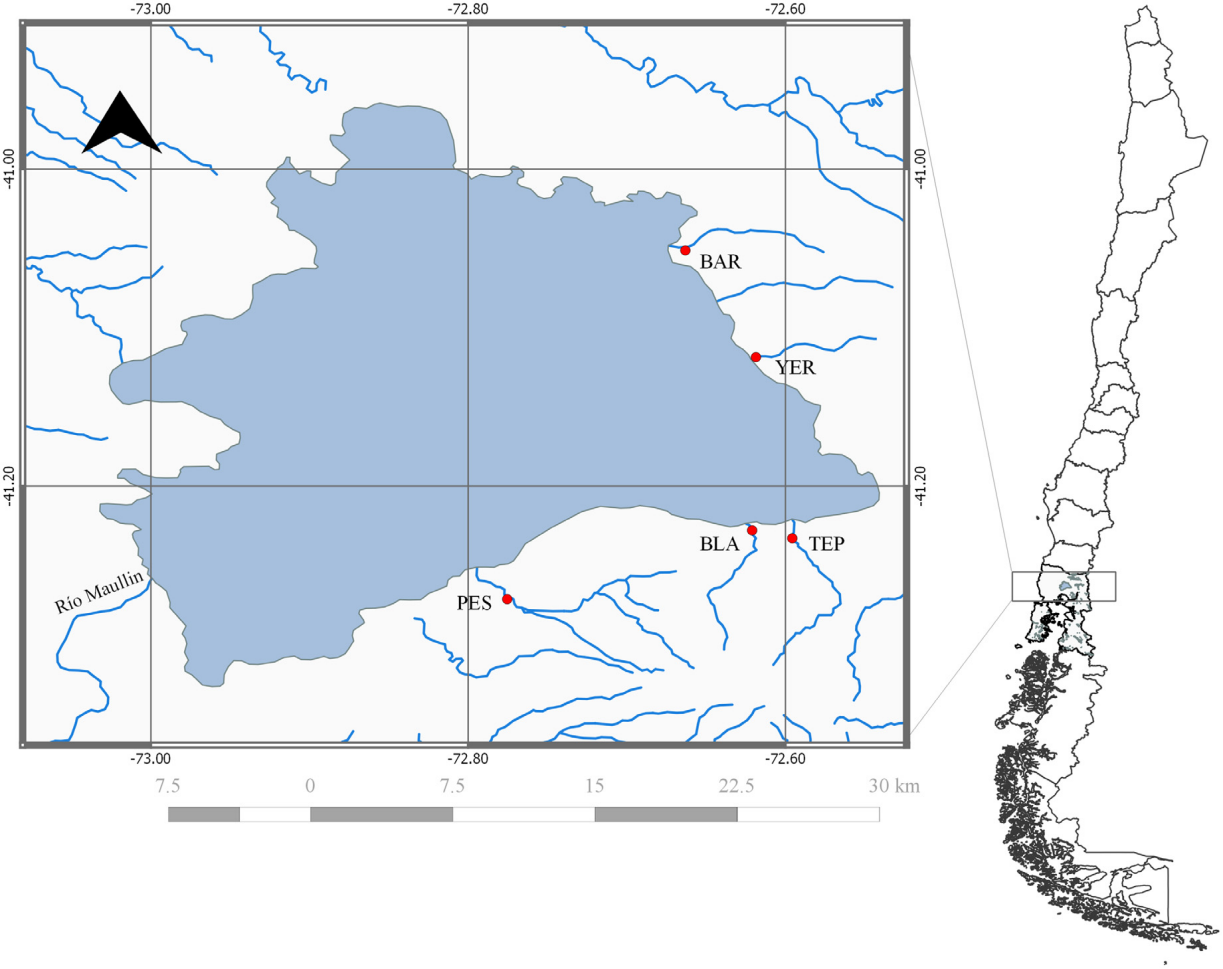
Sampling locations from inlet streams of Lake Llanquihue, Lake District in Chile’s northern Patagonia (from north to south and clockwise): Blanco Arenales (BAR), Yerbas Buenas (YER), Tepu (TEP), Blanco (BLA) y Pescado (PES).

In this study we used population- and individual-based inference from single nucleotide polymorphisms (SNPs) to address three objectives regarding the distribution of genetic variance within and among Rainbow Trout collections from Lake Llanquihue. First, we evaluated intra-population followed by inter-population genetic variance and their statistical significance from collections at five inlet streams off Lake Llanquihue (Fig 1). We hypothesized that intra-population (temporal) genetic variance and temporal instability might be significant among recently founded, historically small, or artificially propagated Rainbow Trout populations similar to that seen in their native range [45,46]. In addition, inter-population divergence might not follow geographic patterns, because invasive fish populations are often in disequilibrium with respect to mutation, migration, and drift [47]. Second, we inferred the putative number of gene pools of Rainbow Trout that might coexist within the lake using individual-based Bayesian inference. Trout of a putative farm origin were identified by typical traits found among farm broodstock, namely skin ulcers or abrasions, short opercula and eroded fins; they are also likely to be genetically different from naturalized trout [17]. Third, we estimated the contemporary number of effective breeders (*N*_*b*_) per year among introduced populations using a linkage disequilibrium method [48]. We hypothesized that estimates of *N*_*b*_ might be small as other invasive Rainbow Trout populations in Patagonia were likely founded two or three generations ago [17]. These hypotheses were tested using multilocus SNP genotypes that were originally ascertained from native Rainbow Trout populations from the west coast of North America [49]. These markers are single base substitutions (mostly biallelic) that are abundant and widespread in the genome, are found in coding and non-coding regions, and can be efficiently genotyped using multiplex PCR screening [50,51]. Our goals promise to contribute to both basic and applied contexts to understand the genetic underpinnings among established populations of a successful invader as well as developing efficient management strategies for invasive species.

## Material and methods

### Study area and sampling design of naturalized populations

Lake Llanquihue (Lake District, X Region: Fig 1) is one of southern Chile’s largest lakes, sharing several characteristics with other Araucanian lakes: (i) a glacial origin with volcanic influences; (ii) a maximum depth greater than 100 m; and (iii) low concentration of nutrients, salts, and chlorophyll-a [52] Lake Llanquihue harbors both native and introduced fish species, primarily salmonids [22]. We surveyed Rainbow Trout in five third-order inlet streams (Fig 1): Blanco Arenales (BAR), Yerbas Buenas (YER), Tepu (TEP), Blanco (BLA) and Pescado (PES). BAR and YER are located in the eastern shore of the lake originating on the slopes of Osorno volcano, at an altitude of approximately at 1000 m [52]. TEP, BLA and PES are located on the southern shore of the lake originating on the slopes of Calbuco volcano, at an altitude ranging from 1000 to 1600 m.

Rainbow Trout collections (N = 582; Table 2) were made using a standard two-pass backpack electrofishing under various settings depending on water conductivity (400–700V; 40–80 Hz). Each inlet stream was sampled twice (or three times in one case: YER) over 13 months in 2012 to 2013 to gauge intra-population (temporal) genetic variance. Sampling occurred during July in austral winter (W) and during October in austral early spring (S), corresponding to the spawning period of Rainbow Trout in Lake Llanquihue [42]. Within each inlet stream, we sampled in all available habitat units (pool-run-riffle) from a 200 m reach using a uniform time effort of 1 h. Collections were labeled as stream plus the last two digits of the year and season (e.g., YER12S).

**Table 2:** Rainbow Trout collections and genetic statistics from inlet streams of Lake Llanquihue.

Inlet stream	Date	Code	Abnormalities	*n*	*H*_*O*_	*H*_*E*_	*A*_*R*_	HWE	*f*	LD *N*_*b*_ (95% CI)
Blanco Arenales (BAR)	Oct 2012	BAR12S	0%	50	0.333	0.335	1.959	0.968	0.017	54 (43–70)
	Jul 2013	BAR13W	0%	44	0.334	0.333	1.984	0.983	0.007	99 (69–163)
Yerbas Buenas (YER)	Oct 2012	YER12S	32%	31	0.365	0.369	1.973	0.955	0.028	26 (21–33)
	Jul 2013	YER13W	0%	33	0.359	0.344	1.951	0.979	0.0273	35 (27–46)
	Oct 2013	YER13S	26%	60	0.368	0.369	1.972	0.896	0.0121	40 (34–48)
Tepu (TEP)	Oct 2012	TEP12S	0%	51	0.323	0.316	1.965	1.000	0.015	135 (86–276)
	Jul 2013	TEP13W	0%	95	0.335	0.322	1.969	0.999	0.035	132 (101–181)
Blanco (BLA)	Oct 2012	BLA12S	0%	49	0.326	0.324	1.977	0.936	0.003	69 (53–95)
	Jul 2013	BLA13W	0%	88	0.312	0.315	1.952	0.967	0.013	121 (92–169)
Pescado (PES)	Oct 2012	PES12S	8%	35	0.332	0.327	1.971	0.904	-0.001	77 (54–131)
	Jul 2013	PES13W	0%	46	0.314	0.308	1.949	1.000	0.009	131 (83–274)

*n*, sample size; *A*_*R*_, allelic richness; *H*_*O*_, observed heterozygosity; *H*_*E*_, expected heterozygosity; HWE, exact probability over multiple loci (Fisher’s method) to test the null hypothesis of Hardy-Weinberg equilibrium proportions; *f*, inbreeding coefficient; LD *N*_*b*_, linkage-disequilibrium estimate for the effective number of breeders.

Fish were anesthetized in a bucket containing a solution of 0.015% v/v of benzocaine (BZ-20^®^; 20% benzocaine) before being photographed, measured, and weighed. We evaluated statistical differences in trout size (total length; cm) from different streams (and groups thereof) using non-parametric Mann-Whitney *U* tests in R [53]. We recorded abnormalities such as eroded fins and short opercula as these might suggest a farm broodstock origin [17]. Fin clips of 25–100 mm^2^ in size from the adipose fin, caudal fin, or both, were non-lethally obtained and preserved in ethanol 95%. Fish were put in a recovery tank containing well oxygenated water before release.

### SNP genotyping

Genomic DNA was isolated from fin clips using a Qiagen DNeasy Tissue Kit (Valencia, California, USA) following the manufacturer’s instructions. We used a panel of 96 polymorphic SNPs [54,55] which were originally ascertained from several populations spanning the native range of Rainbow Trout [49]. Multiplex PCR was carried out using Fluidigm^®^ 96.96 dynamic array chips following established pre-amplification and multiplex PCR protocols [50,56].

### SNP Selection

Prior to estimate genetic diversity within and among naturalized samples, we implemented BAYESCAN [57] to identify putative candidate SNPs that might be influenced by natural selection. We tested for deviations from Hardy-Weinberg equilibrium (HWE) and for linkage disequilibrium (LD: non-random association of alleles between loci) for all SNPs in each collection site using GENEPOP[58].

### Genetic diversity within collections

We used GENALEX to estimate observed (*H*_*O*_) and expected heterozygosities (*H*_*E*_) within naturalized collections. Allelic richness (*A*_*R*_) and inbreeding coefficients (*f*) were calculated in FSTAT [59]. To compare genetic diversity among collections (or groups thereof) we used permutational multivariate analysis of variance (PERMANOVA) implemented in the package *vegan* in R [53]. The program estimates Euclidean distances between observed and expected values following 10,000 permutations, and then tests the null hypothesis of no differences between observed and simulated distance matrices.

### Intra- and inter-population genetic variance

We calculated intra-population *θ* (between temporal replicates within streams) as well as inter-population *θ* (among streams) using GENEPOP [58,60]. To test if *θ* were significantly greater than zero, we estimated chi-square probabilities in CHIFISH [61]. We implemented an analysis of molecular variance (AMOVA: [62]) to evaluate the significance of intra- vs. inter-population genetic variances following 10,000 permutations of multilocus genotypes using GENALEX. Hierarchical components of genetic variance and their significance were reported using sums of squares and *F* statistics.

### Individual-based Bayesian inference of genetic structure

We used Bayesian inference to assign individual genotypes to a defined number of genetic pools (*K*) in STRUCTURE [63,64]. The program probabilistically assigns individual multilocus genotypes into a discrete number of clusters while minimizing departures from HWE and LD from admixed populations. We performed separate simulations for spring and winter sampling seasons. We ran 15 iterations per each *K*, which ranged between one and five using an admixture model and a burn-in period of 50,000, followed by 250,000 Markov Chain Monte Carlo steps after burn-in. To evaluate the most probable value for *K*, we followed the Evanno criterion [65] using STRUCTURE HARVESTER [66]. We plotted ‘consensus’ coefficients of individual membership (*Q*-values) in R following cluster matching and permutation in CLUMPP [67] to account for label switching artifacts and multimodality.

### Estimation of the effective numbers of breeders (*N*_*b*_)

We estimated the contemporary annual number of effective breeders (*N*_*b*_) in naturalized collections using LDNE [48]. The method evaluates LD between unlinked loci as a *proxy* for genetic drift assuming selective neutrality, discrete generations, and closed populations. While the first might be met, the second might not as salmonid populations are age-structured and are likely to be connected by gene flow. When applied to age-structured populations, LD reflects a quantity closer to *N*_*b*_ per brood year rather than the effective population size per generation [38]. The method seems robust to equilibrium gene flow, unless migration rates are higher than 5–10% [68]. LDNE was run with the following settings: (i) minor allele frequencies < 0.02 were excluded to account for the trade-off between accuracy and precision at sample sizes larger than 25 individuals per collection [38], (ii) random mating system, and (iii) 95% CIs were calculated via jackknifing of LD values among pairs of loci. Comparisons of *N*_*b*_ between groups of naturalized trout were performed through non-parametric Mann-Whitney tests in R.

### Ethics statement

This study was carried out in accordance with the recommendations of the Guidelines for the Use of Fishes in Research (http://fisheries.org/guide-for-the-use-of-fishes-in-research). The protocol was approved by the Committee on the Ethics of Animal Experiments of the Universidad de Concepcion. Fishing permit #1715/14 was issued by the Chilean Undersecretary of Fisheries and Aquaculture.

## Results

### Sampling: variation in fish size and evidence of farmed trout

**Fig 2:**
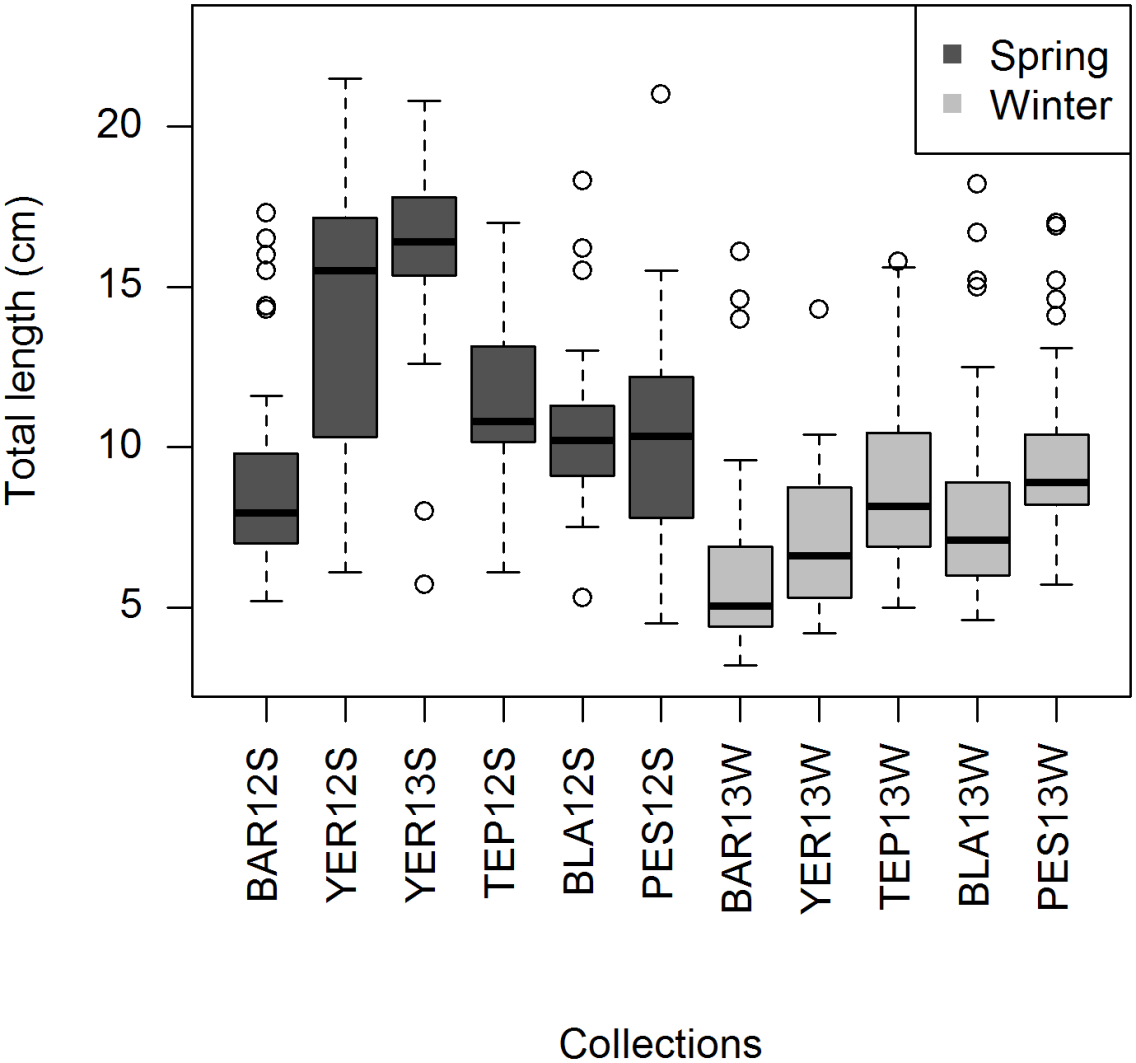
Boxplots of Rainbow Trout size (total length, TL; cm) among streams collections from Lake Llanquihue during spring and winter.

**Fig 3:**
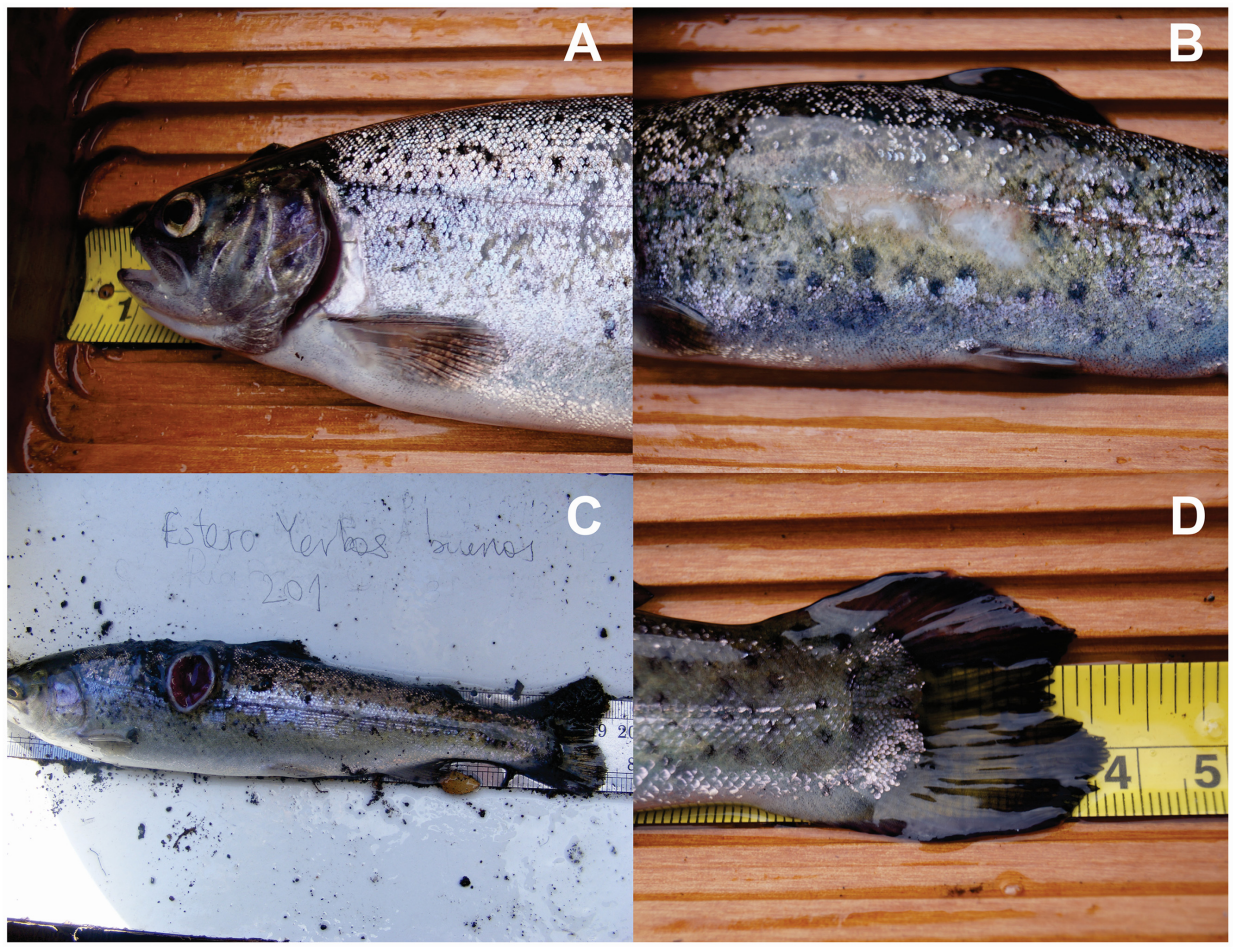
Examples of abnormalities found in Rainbow Trout collected from inlet streams of Lake Llanquihue: A) short opercula, B) skin abrasions, C) skin ulcer, and D) eroded fin.

Rainbow Trout from inlet streams of Lake Llanquihue varied in size from 5 to 25 cm TL with a median below 15 cm in most collections (Fig 2). We found significant differences in fish size between spring and winter collections; spring trout were larger than winter trout across collections (Mann-Whitney *U* test: *U* = 45646, *p* < 0.001). Also, trout from YER12S and YER13S were larger than trout caught at any of the remaining streams during spring (Mann-Whitney *U* test: *U* = 10098: *p* ˂ 0.001). Further, collections PES12S, YER12S and YER13S showed trout with skin ulcers, skin abrasions, eroded fins, and short opercula, suggestive of farmed fish (Fig 3). The percentage of trout showing these characteristics varied between 8% (PES12S) and 32% (YER13S: Table 2).

### SNP selection

SNPs were removed if they were monomorphic in all populations, consistently deviated from HWE proportions, showed evidence of directional selection, or exhibited significant LD (S1 Table). Three monomorphic loci were dropped from the analyses: *Ocl_Okerca*, *Ocl_oku202* and *Ocl_Oku216*. We found consistent deviations from HWE proportions across populations in *Omy_mcsf-268*, *Omy_09AAD-076*, and *Omy_110064–419* and removed these from the data set. A putative candidate for directional selection was found in locus *Omy_107806–34* which was excluded. We found significant LD (*p* < 0.001) in more than 75% of collections (often in 100% of collections) for five groups of loci, some of which had known linkage relationships from previous studies [49,69] as presented in S1 Table. Additional linkage information was found using BLAST against contigs from whole genome sequencing of Rainbow Trout [70]. Significant matches and close proximity between SNPs suggested that LD was likely the result of physical linkage between markers (S1 Table). We thus kept the most informative of each group as suggested elsewhere [71]. We found no further evidence for deviations from either HWE proportions or LD within populations after removal of affected loci. The filtering process left a final panel of 81 SNPs for subsequent analyses.

### Genetic diversity within collections

We estimated values of *H*_*O*_ between 0.312 (BLA13W) and 0.368 (YER13S), whereas values of *H*_*E*_ ranged between 0.315 (BLA13W) and 0.369 (YER12S, YER13S; Table 2). *A*_*R*_ varied between 1.951 (YER13W) and 1.984 (BAR13W). Trout from YER had higher *H*_*E*_ than trout from any of the remaining collections (PERMANOVA: *p* < 0.001).

### Intra- and inter-population genetic variance

We found significant intra- and inter-population divergence measured as *θ* among all collections with only three exceptions—BAR12 S vs. PES12S, BLA12S vs. BAR13W, and PES12S vs. BLA13W (Table 3). Significant *θ* values ranged between 0.091 (TEP12S vs. YER13S) and 0.004 (PES13W vs. BLA13W) and did not follow simple geographic patterns mediated by distance. For instance, *θ* values between inlet streams separated by less than 5 km (e.g., BLA12S vs. TEP12S) were larger than between inlet streams separated by more than 30 km (e.g., PES12 vs. BAR12). The highest *θ* values were found in inter-population pairwise comparisons involving all three YER collections. Hierarchical AMOVA suggested that both intra- and inter-population genetic variance were significant, and the latter was higher than the former if all collections were included in the analysis (Table 4). However, intra-population variance was higher than inter-population variance (which was marginally significant: 0.01 ˂ p ˂ 0.05) after excluding highly distinct YER collections.

**Table 3:** Pairwise genetic distances *(θ*) between collections. Probabilities^a^ from χ^2^ tests for the null hypothesis of no differentiation at any locus are shown next to *θ* values.

Collection	BAR12S	BAR13W	YER12S	YER13S	YER13W	TEP12S	TEP13W	BLA12S	BLA13W	PES12S
BAR13W	0.0040**									
YER12S	0.0530**	0.0527**								
YER13S	0.0585**	0.0630**	0.0156**							
YER13W	0.0181**	0.0166**	0.0287**	0.0459**						
TEP12S	0.0073**	0.0116**	0.075**	0.0911**	0.0330**					
TEP13W	0.0057**	0.0074**	0.0669**	0.0762**	0.0282**	0.0049**				
BLA12S	0.0066**	**0.0032**	0.0657**	0.0743**	0.0220**	0.0128**	0.0088**			
BLA13W	0.0066*	0.0079**	0.0768**	0.0843**	0.0285**	0.0072**	0.0076**	0.0065**		
PES12S	**0.0022**	0.0059*	0.0663**	0.0785**	0.0245**	0.0046*	0.0039*	0.0054*	**0.0014**	
PES13W	0.0113**	0.0076**	0.0810**	0.0867**	0.0288**	0.0111**	0.0103**	0.0103**	0.0035*	0.0048*

^a^ *p* < 0.01

*, *p* < 0.001

**, non-significant in bold.

**Table 4:** Hierarchical analysis of molecular variance (AMOVA) for spatial and temporal components.

Source of variation	df	SS	% Variance	F-statistic	p-value
**All collections**					
Inter-population	4	379.4	2%	0.021	0.001
Intra-population	5	115.2	1%	0.027	0.001
**Excluding YER collections** ^a^					
Inter-population	3	85.0	0%	0.002	0.048
Intra-population	4	85.9	1%	0.007	0.001

df, degrees of freedom; SS, sums of squares.

^a^YER12S, YER13S and YER13W.

### Individual-based Bayesian clustering: spring vs. winter genetic structure

**Fig 4:**
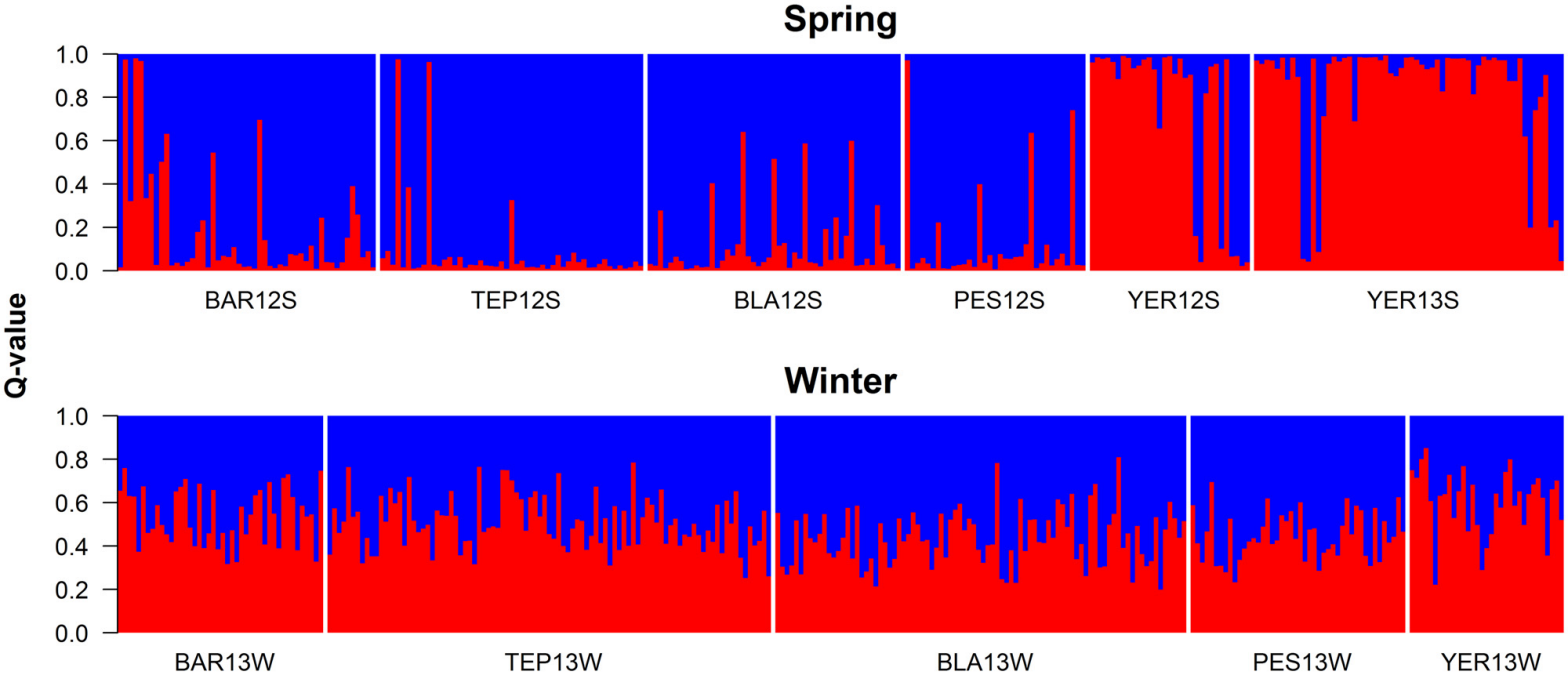
Individual ancestry coefficients (*Q*-values) among Rainbow Trout genotypes collected from inlet streams of Lake Llanquihue when assigned to two gene pools (*K* = 2) during spring or winter.

The number of gene pools inferred through Bayesian clustering varied depending on sampling season (Fig 4). We found evidence for *K* = 2 during spring; asymmetric *Q*-values from trout sampled from from YER12S and YER13S suggested a unique ancestry in comparison to other collections. However, we found no clear evidence for genetic structure during winter (*K* = 1). *Q*-values among collections were relatively symmetric (0.45–0.55) when assigned to two clusters, with exception of YER13W that showed *Q*-values between 0.39 and 0.61.

### Estimation of the effective numbers of breeders (*N*_*b*_)

All estimates of *N*_*b*_ were bound by finite 95% CIs, suggesting that the number of annual breeders contributing to all collections might be small (Table 2). Point estimates were below 150 and the median *N*_*b*_ was 73. Estimates of *N*_*b*_ for YER12S, YER13S and YER13W were the smallest and significantly lower than any other collections (Mann-Whitney *U* test: *U* = 0, *p* = 0.008).

## Discussion

We used population- and individual-based inference of multilocus SNP genotypes to address three fundamental issues of the naturalization and invasion of Rainbow Trout in a lake historically impacted by trout stocking and farming practices. First, we found significant intra- and inter-population genetic variance, and the former (temporal) component seemed more important than the latter (spatial) component if highly distinct collections were excluded from the analyses. Spatial differentiation as revealed by estimates of inter-population *θ* did not follow simple geographic patterns mediated by distance. Second, individual-based Bayesian analyses further revealed that trout from one single stream, Yerbas Buenas (YER), drove genetic divergence among individuals during spring, but not in winter sampling seasons. Spring trout from YER were additionally more likely to show abnormalities and have smaller *N*_*b*_ than trout from any other collection, suggesting they might originate from farm broodstock. Third, our estimates of *N*_*b*_ showed that half of the collections had a breeding size of 73, consistent with previous findings of significant temporal genetic variance. We discuss these findings in detail in the following paragraphs.

### Intra- and inter-population genetic variance

With exception of Pink Salmon where temporal divergence is prominent between odd- and even-year populations, spatial divergence at various scales as a result of homing behavior is a trademark among salmonid systems in their native range [72,73], usually surpassing the magnitude of temporal divergence in large, wild populations in pristine environments (Pacific salmon: [44,74]; Atlantic salmon, *Salmo salar*: [75,76]; brown trout, *S*. *trutta*: [77]). For naturalized Rainbow Trout from Lake Llanquihue, pairwise *θ* showed that intra- population (temporal) genetic variance was greater than inter-population (spatial) genetic variance, downplaying the importance of spatial divergence during the process of naturalization.

What is the explanation behind significant temporal genetic variance? We ruled out sources of confounding spatiotemporal effects such as family-biased sampling as no significant departures from HWE proportions or LD were evident within collections. One hypothesis is that winter and spring trout collections correspond to different cohorts and that genetic drift between cohorts was significantly higher than expected by chance. This is consistent with differences in size (and likely age) between spring and winter trout. Most spring trout likely comprised fish of age +1 (i.e., fish that hatched in late spring and spent one winter) and or even age +2 (e.g., YER12S and YER 13S), whereas winter trout likely comprised young-of-the-year fish [42]. Thus, differences in allele frequencies between cohorts might be an indication of a reduced number of annual breeders (small *N*_*b*_).

Our findings are limited to collections temporally spaced 8 to 13 months and they need not reflect patterns of genetic drift at longer temporal scales. Rainbow Trout of ages 4+ to 12+ (with a mode at age 6+) breeding at the same inlet streams we examined here (PES, TEP and BLA) were described in an earlier study, suggesting an iteroparous life history [42]. Multiple breeding seasons among Rainbow Trout from Lake Llanquihue imply that genetic monitoring over longer time scales—assuming one generation occurs every five years—should be accomplished in order to appropriately assess inter-generational changes, or alternatively, estimating temporal changes from cohort-based approaches to populations with overlapping generations [78].

Lastly, significant inter-population *θ* which was nearly universal among all collections and lack of geographic structure should be interpreted within the context of invasive populations that might not be at migration-drift equilibrium [47]. This is a likely scenario for Rainbow Trout in Chile’s Lake District [17] and Lake Llanquihue, wherein populations might have established eight to twenty generations ago, assuming a generation time of five years [42]. Estimates of *θ* will fail to translate into realistic measures of gene flow if populations are in disequilibrium [37], which argues for the application of individual-based approaches to understand genetic structure and connectivity among invasive populations [35,36].

### Individual-based Bayesian clustering: propagule-driven genetic structure

We identified two gene pools of Rainbow Trout coexisting in Lake Llanquihue during spring, but only one during winter sampling seasons. During spring, one of these pools comprised trout populating one single stream: Yerbas Buenas (YER), a narrow (1–2 m wetted channel width), fast-flowing (approximately 1 m s^-1^), and one of the most productive inlet streams for Rainbow Trout (authors’ unpublished data). We speculate that the source of YER trout appears to be farm broodstock that migrated upstream to breed in spring, which explains why during winter they were apparently missing; it is beyond the scope of our study to determine whether YER12S and YER13S originated from one or two consecutive escaping events. Farm escapees are more likely to show abnormalities and disease that affect farmed trout [17,79]; they also had the highest heterozygosity and the smallest estimates of *N*_*b*_, suggesting a highly diverse and yet genetically small source. A common disease affecting Rainbow Trout in freshwater is flavobacteriosis (*Flavobacterium psychrophilum*) with characteristic skin ulcers and erosions as reported here (R. Avendaño, comm. pers. 2015). These results are stimulating because farm propagules of Rainbow Trout can not only be identified and distinguished from naturalized individuals; they might establish in the wild and breed with other farmed or naturalized trout. Indeed, we observed several sexually mature trout from YER during spring (authors’ field observations), suggesting a time of breeding that begins in winter but extends into early spring [42].

While identifying farm escapees can be accomplished using other methods [80], SNP genotyping might be a faster and more cost-effective method to discriminate between naturalized and farmed trout than other genetic approaches [81]. Further work should be aimed at genotyping farm broodstock from this lake and appraise their similarity with YER collections using individual assignment, to confirm our hypothesis. Such data will also help clarify if farm broodstock might have interbred with naturalized trout in YER and other streams by identifying farm-naturalized hybrids and their abundance. This is an interesting possibility and might explain why YER13W juveniles showed *Q*-values that departed slightly from “pure” naturalized trout found among remaining collections during winter that were symmetrically assigned to two clusters. Consuegra et al. [17] genetically characterized farmed-naturalized Rainbow Trout hybrids which were more abundant near farms, arguing that propagule-driven admixture might have facilitated establishment of Rainbow Trout in a large portion of the Lake District in southern Chile. Rainbow Trout has become the most abundant fish species at populating streams of Lake Llanquihue, displacing not only native fishes but other non-native salmonids including Brown Trout (*Salmo trutta*) and Coho Salmon (*Oncorhynchus kisutch*), which are more conspicuous in lakes with low or no propagule pressure from aquaculture farms [22,24]. Our findings argue for efficient management measures that can mitigate Rainbow Trout escapes in freshwater given that they hold a great potential to establish self-sustaining populations and spread of the invasion [16].

### Estimation of the effective numbers of breeders (*N*_*b*_)

Our estimates of *N*_*b*_ suggest that half of naturalized trout collections have an annual breeding size of 73 individuals or less, which appears counterintuitive given their high levels of heterozygosity. This supports the notion that these populations might be experiencing substantial genetic drift and temporal genetic variance. Parallel estimates of LD *N*_*b*_ for Rainbow Trout populations in Patagonia were below 100 based on microsatellite DNA multilocus genotypes [17]. This is interesting because *H*_*E*_ among naturalized collections remained high despite small LD *N*_*b*_. We performed a quick comparison with native populations from North America (n = 10) [49] genotyped for matching SNPs and found that naturalized trout in Lake Llanquihue were more heterozygous than trout from ten native populations (PERMANOVA: *p* < 0.001; S1 Fig). We hypothesize that genetic diversity has remained high due to historical, rather than recent, admixture as measures of heterozygosity are generally unaffected during bottlenecks [82,83]. High *H*_*E*_ is also consistent with multiple “waves” of introduction in Lake Llanquihue likely originating from genetically distinct sources, a possible explanation of why invasive populations generally overcome founding effects and spread [84].

The paradox of genetically diverse but small-size populations might not be exclusive of Rainbow Trout outside their native range and might suggest recently founded populations [17]. Native steelhead populations (anadromous life history of Rainbow Trout) from the Skeena River in British Columbia (Canada) showed estimates of *N*_*b*_ below 100 in most cases, despite the fact of census population sizes (*N*) in the order of 1000s [46]. To our knowledge, reliable estimates of *N* are unavailable for inlet streams of Lake Llanquihue, and these are necessary to comprehend ecological processes affecting these populations. In addition, the ratio *N*_*b*_/*N* is crucial to assess population stochasticity in the face of recent global environmental change [39]. This theoretical framework, conceived and often applied to native populations, can be extended to invasive populations and their management and control, for instance, to predict which populations are more likely to persist [47,85,86]. Lastly, our survey serendipitously offers a good baseline for monitoring the ecological and demographic consequences of the eruption of Calbuco volcano on aquatic communities [87], which deposited large quantities of volcanic ash on inlet streams TEP, BLA and PES.

## Conclusions

Using population- and individual-based inference of SNP multilocus genotypes we provide insights on the genetic influence on the establishment and naturalization of Rainbow Trout in Lake Llanquihue, a Patagonian lake heavily affected by trout farming. First, we found that intra- population (temporal) genetic variance was greater than inter-population (spatial) genetic variance, downplaying the importance of spatial divergence during the process of naturalization. Temporal variance might be explained by allele frequency shifts between cohorts, consistent with variation in fish length between spring and winter collections. Second, individual-based Bayesian clustering suggested that genetic structure within Lake Llanquihue has been largely influenced by putative farm propagules from Yerbas Buenas (YER) stream. Yet, it is unclear whether interbreeding has occurred between “pure” naturalized and farm trout. Third, estimates of the annual number of breeders (*N*_*b*_) were below 73 in half of the five collections, suggesting genetically small and recently founded populations that might experience substantial genetic drift. Our findings reinforce the notion that naturalized trout originated recently from a small yet genetically diverse source and that farm propagules might have played a significant role in the invasion of Rainbow Trout within a single lake.

## Supporting Information

S1 Fig Expected heterozygosities among Lake Llanquihue’s naturalized trout and North America’s native rainbow trout and steelhead populations genotyped for identical SNPs. (TIF)

S1 Table List of SNP loci used in the study. Linkage information between loci is emphasized using lower case letters.
(XLSX)

## References

[1] Pysek P, Richardson DM. Invasive Species, Environmental Change and Management, and Health In: Gadgil A, Liverman DM, editors. Annual Review of Environment and Resources, Vol 35. Annual Review of Environment and Resources. Palo Alto: Annual Reviews; 2010 p. 25–55.

[2] Mack RN, Simberloff D, Lonsdale WM, Evans H, Clout M, Bazzaz FA. Biotic invasions: Causes, epidemiology, global consequences, and control. Ecological Applications. 2000;10(3):689–710. 10.2307/2641039 WOS:000087506600008.

[3] Simberloff D. The Role of Propagule Pressure in Biological Invasions. Annual Review of Ecology Evolution and Systematics. 2009;40:81–102. 10.1146/annurev.ecolsys.110308.120304 WOS:000272455700005.

[4] Kolar CS, Lodge DM. Progress in invasion biology: predicting invaders. Trends in Ecology & Evolution. 2001;16(4):199–204. 11245943 10.1016/s0169-5347(01)02101-2

[5] Marchetti MP, Moyle PB, Levine R. Invasive species profiling? Exploring the characteristics of non-native fishes across invasion stages in California. Freshwater Biology. 2004;49(5):646–61.

[6] Garcia-Berthou E. The characteristics of invasive fishes: what has been learned so far? Journal of Fish Biology. 2007;71:33–55. 10.1111/j.1095-8649.2007.01668.x WOS:000252333000002.

[7] Pascual MA, Cussac V, Dyer B, Soto D, Vigliano P, Ortubay S, et al Freshwater fishes of Patagonia in the 21st Century after a hundred years of human settlement, species introductions, and environmental change. Aquatic Ecosystem Health & Management. 2007;10(2):212–27.

[8] De Leaniz CG, Gajardo G, Consuegra S. From Best to Pest: changing perspectives on the impact of exotic salmonids in the southern hemisphere. Systematics and Biodiversity. 2010;8(4):447–59. 10.1080/14772000.2010.537706 ISI:000285516200005.

[9] Pascual MA, Lancelotti JL, Ernst B, Ciancio JE, Aedo E, Garcia-Asorey M. Scale, connectivity, and incentives in the introduction and management of non-native species: the case of exotic salmonids in Patagonia. Frontiers in Ecology and the Environment. 2009;7(10):533–40. 10.1890/070127 WOS:000272245000016.

[10] Aqua. Peligran las siembras de salmones en el Lago Calafquén. [Digital]. http://www.aqua.cl/2007/05/22/peligran-las-siembras-de-salmones-en-el-lago-calafquen/:

[11] Perez C. Skretting participa en proyecto de repoblamiento de lagos http://www.salmonexpert.cl/index.php?article_id=109597: Salmon Expert; 2014 [22 April 2015].

[12] Garcia Alvarado M. Bases Técnicas para establecer Reglamento de Siembra y Repoblación con fines de Pesca Recreativa. http://www.subpesca.cl/publicaciones/606/articles-76266_documento.pdf :Subsecretaria de Pesca y Acuicultura, 2008 Contract No.: 57/2008.

[13] Basulto S. El Largo viaje de los salmones: una crónica olvidada, Propagación y cultivo de especies acuáticas en Chile. Santiago de Chile: Editorial Maval, Ltda; 2003 299 p.

[14] Buschmann AH, Cabello F, Young K, Carvajal J, Varela DA, Henriquez L. Salmon aquaculture and coastal ecosystem health in Chile: Analysis of regulations, environmental impacts and bioremediation systems. Ocean & Coastal Management. 2009;52(5):243–9.

[15] León-Muñoz J, Teckin D, Farias A, Diaz S. Salmon Farming in the Lakes of Southern Chile—Valdivian Ecoregion. WWF & Núcleo Científico Milenio Forecos, Universidad Austral de Chile, 2007.

[16] Sepulveda M, Arismendi I, Soto D, Jara F, Farias F. Escaped farmed salmon and trout in Chile: incidence, impacts, and the need for an ecosystem view. Aquaculture Environment Interactions. 2013;4:273–83.

[17] Consuegra S, Phillips N, Gajardo G, de Leaniz CG. Winning the invasion roulette: escapes from fish farms increase admixture and facilitate establishment of non-native rainbow trout. Evolutionary Applications. 2011;4(5):660–71. 10.1111/j.1752-4571.2011.00189.x WOS:000294924900005. 25568013 PMC3352532

[18] Monzon-Arguello C, Consuegra S, Gajardo G, Marco-Rius F, Fowler DM, DeFaveri J, et al Contrasting patterns of genetic and phenotypic differentiation in two invasive salmonids in the southern hemisphere. Evolutionary Applications. 2014;7(8):921–36. 10.1111/Eva.12188 ISI:000342756300007. 25469171 PMC4211722

[19] Monzon-Arguello C, de Leaniz CG, Gajardo G, Consuegra S. Eco-immunology of fish invasions: the role of MHC variation. Immunogenetics. 2014;66(6):393–402. 10.1007/s00251-014-0771-8 ISI:000336270000004. 24752816

[20] Monzon-Arguello C, de Leaniz CG, Gajardo G, Consuegra S. Less can be more: loss of MHC functional diversity can reflect adaptation to novel conditions during fish invasions. Ecology and Evolution. 2013;3(10):3359–68. 24223274 10.1002/ece3.701 PMC3797483

[21] Correa C, Hendry AP. Invasive salmonids and lake order interact in the decline of puye grande Galaxias platei in western Patagonia lakes. Ecological Applications. 2012;22(3):828–42. WOS:000303312000008. 22645814 10.1890/11-1174.1

[22] Soto D, Arismendi I, Gonzalez J, Sanzana J, Jara F, Jara C, et al Southern Chile, trout and salmon country: invasion patterns and threats for native species. Revista Chilena De Historia Natural. 2006;79(1):97–117. WOS:000236490000009.

[23] Soto D, Jara F, Moreno C. Escaped salmon in the inner seas, southern Chile: Facing ecological and social conflicts. Ecological Applications. 2001;11(6):1750–62. 10.2307/3061093 WOS:000172456700015.

[24] Arismendi I, Soto D, Penaluna B, Jara C, Leal C, Leon-Munoz J. Aquaculture, non-native salmonid invasions and associated declines of native fishes in Northern Patagonian lakes. Freshwater Biology. 2009;54(5):1135–47. 10.1111/j.1365-2427.2008.02157.x WOS:000265012000016.

[25] Habit E, Gonzalez J, Ruzzante DE, Walde SJ. Native and introduced fish species richness in Chilean Patagonian lakes: inferences on invasion mechanisms using salmonid-free lakes. Diversity and Distributions. 2012;18(12):1153–65. 10.1111/j.1472-4642.2012.00906.x WOS:000310727500001.

[26] Habit E, Piedra P, Ruzzante DE, Walde SJ, Belk MC, Cussac VE, et al Changes in the distribution of native fishes in response to introduced species and other anthropogenic effects. Global Ecology and Biogeography. 2010;19(5):697–710. 10.1111/j.1466-8238.2010.00541.x WOS:000280633800010.

[27] Vargas PV, Arismendi I, Lara G, Millar J, Peredo S. Evidence of microhabitat overlap between juvenile of introduced salmon *Oncorhynchus tshawytscha* and the native fish *Trichomycterus areolatus* in the Allipen River, Chile. Revista De Biologia Marina Y Oceanografia. 2010;45(2):285–92. WOS:000281186300010.

[28] Barrett SCH. Foundations of invasion genetics: the Baker and Stebbins legacy. Molecular Ecology. 2014:n/a–n/a. 10.1111/mec.13014 25442107

[29] Dlugosch KM, Parker IM. Founding events in species invasions: genetic variation, adaptive evolution, and the role of multiple introductions. Molecular Ecology. 2008;17(1):431–49. 10.1111/j.1365-294X.2007.03538.x WOS:000251740500035. 17908213

[30] Keller SR, Taylor DR. Genomic admixture increases fitness during a biological invasion. Journal of Evolutionary Biology. 2010;23(8):1720–31. 10.1111/j.1420-9101.2010.02037.x WOS:000279901400015. 20626546

[31] Becker LA, Pascual MA, Basso NG. Colonization of the southern Patagonia ocean by exotic Chinook salmon. Conservation Biology. 2007;21(5):1347–52. 10.1111/j.1523-1739.2007.00761.x WOS:000250008700026. 17883500

[32] Riva-Rossi CM, Pascual MA, Marchant EA, Basso N, Ciancio JE, Mezga B, et al The invasion of Patagonia by Chinook salmon (Oncorhynchus tshawytscha): inferences from mitochondrial DNA patterns. Genetica. 2012;140(10–12):439–53. 10.1007/s10709-012-9692-3 WOS:000314333200004. 23188114

[33] Præbel K, Gjelland KØ, Salonen E, Amundsen P-A. Invasion genetics of vendace (Coregonus albula (L.)) in the Inari-Pasvik watercourse: revealing the origin and expansion pattern of a rapid colonization event. Ecology and Evolution. 2013;3(5):1400–12. 10.1002/ece3.552 23762524 PMC3678492

[34] Rollins LA, Moles AT, Lam S, Buitenwerf R, Buswell JM, Brandenburger CR, et al High genetic diversity is not essential for successful introduction. Ecology and Evolution. 2013;3(13):4501–17. 10.1002/Ece3.824 ISI:000326824300019. 24340190 PMC3856749

[35] Herborg LM, Weetman D, Van Oosterhout C, Hanfling B. Genetic population structure and contemporary dispersal patterns of a recent European invader, the Chinese mitten crab, *Eriocheir sinensis* . Molecular Ecology. 2007;16(2):231–42. ISI:000243305200001. 17217341 10.1111/j.1365-294X.2006.03133.x

[36] Fraser EJ, Macdonald DW, Oliver MK, Piertney S, Lambin X. Using population genetic structure of an invasive mammal to target control efforts—An example of the American mink in Scotland. Biological Conservation. 2013;167:35–42. 10.1016/j.biocon.2013.07.011 ISI:000328804300005.

[37] Whitlock MC, McCauley DE. Indirect measures of gene flow and migration: F-ST not equal 1/(4Nm+1). HEREDITY. 1999;82:117–25. ISI:000079147200001. 10098262 10.1038/sj.hdy.6884960

[38] Waples RS, Do C. Linkage disequilibrium estimates of contemporary N-e using highly variable genetic markers: a largely untapped resource for applied conservation and evolution. Evolutionary Applications. 2010;3(3):244–62. 10.1111/j.1752-4571.2009.00104.x ISI:000276790700002. 25567922 PMC3352464

[39] Palstra FP, Fraser DJ. Effective/census population size ratio estimation: a compendium and appraisal. Ecology and Evolution. 2012;2(9):2357–65. 10.1002/Ece3.329 ISI:000312449300024. 23139893 PMC3488685

[40] Lowe S BM, Boudjelas S, De Poorter M 100 of the World’s Worst Invasive Alien Species, 2nd edn (IUCN) TISSGIasgotSSCSotWCU, editor. Auckland, New Zealand 2000 12 p.

[41] Arismendi I, Penaluna BE, Dunham JB, de Leaniz CG, Soto D, Fleming IA, et al Differential invasion success of salmonids in southern Chile: patterns and hypotheses. Reviews in Fish Biology and Fisheries. 2014;24(3):919–41. 10.1007/s11160-014-9351-0 ISI:000340455200013.

[42] Arismendi I, Sanzana J, Soto D. Seasonal age distributions and maturity stage in a naturalized rainbow trout (Oncorhynchus mykiss Walbaum) population in southern Chile reveal an ad-fluvial life history. Annales De Limnologie-International Journal of Limnology. 2011;47(2):133–40. 10.1051/limn/2011012 WOS:000292876800003.

[43] Hendry AP, Wenburg JK, Bentzen P, Volk EC, Quinn TP. Rapid evolution of reproductive isolation in the wild: Evidence from introduced salmon. Science. 2000;290(5491):516–8. ISI:000089946000051. 11039932 10.1126/science.290.5491.516

[44] Gomez-Uchida D, Seeb JE, Habicht C, Seeb LW. Allele frequency stability in large, wild exploited populations over multiple generations: insights from Alaska sockeye salmon (Oncorhynchus nerka). Canadian Journal of Fisheries and Aquatic Sciences. 2012;69(5):916–29. 10.1139/f2012-029 WOS:000303447800011.

[45] Blankenship SM, Small MP, Bumgarner JD. Temporal Stability of Genetic Variation within Natural Populations of Summer Steelhead Receiving Mitigation Hatchery Fish. Transactions of the American Fisheries Society. 2009;138(5):1052–64. 10.1577/t08-165.1 ISI:000272025600011.

[46] Heath DD, Busch C, Kelly J, Atagi DY. Temporal change in genetic structure and effective population size in steelhead trout (Oncorhynchus mykiss). Molecular Ecology. 2002;11(2):197–214. ISI:000173726200005. 11856422 10.1046/j.1365-294x.2002.01434.x

[47] Hanfling B. Understanding the establishment success of non-indigenous fishes: lessons from population genetics. Journal of Fish Biology. 2007;71:115–35. 10.1111/j.1095-8649.2007.01685.x WOS:000252333000007.

[48] Waples RS, Do C. LDNE: a program for estimating effective population size from data on linkage disequilibrium. Molecular Ecology Resources. 2008;8(4):753–6. 10.1111/j.1755-0998.2007.02061.x ISI:000257511600005. 21585883

[49] Limborg MT, Blankenship SM, Young SF, Utter FM, Seeb LW, Hansen MHH, et al Signatures of natural selection among lineages and habitats in *Oncorhynchus mykiss* . Ecology and Evolution. 2012;2(1):1–18. 10.1002/ece3.59 WOS:000312442000001. 22408722 PMC3297173

[50] Seeb JE, Pascal CE, Ramakrishnan R, Seeb LW. SNP Genotyping by the 5'-Nuclease Reaction: Advances in High-Throughput Genotyping with Nonmodel Organisms. In: Komar AA, editor. Single Nucleotide Polymorphisms, Methods in Molecular Biology 2009. 10.1007/978-1-60327-411-1_18 19768601

[51] Helyar SJ, Hemmer-Hansen J, Bekkevold D, Taylor MI, Ogden R, Limborg MT, et al Application of SNPs for population genetics of nonmodel organisms: new opportunities and challenges. Molecular Ecology Resources. 2011;11:123–36. 10.1111/j.1755-0998.2010.02943.x WOS:000287485100012. 21429169

[52] Campos H, Steffen W, Agüero G, Parra O, Zúñiga L. Limnological study of Lake Llanquihue (Chile): morphometry, physics, chemistry, plankton and primary productivity. Arch Hydrobiol, Suppl. 1988;81:37–67.

[53] R_Development_Core_Team. R: A language and environment for statistical computing. R Foundation for Statistical Computing, Vienna, Austria ISBN 3-900051-07-0, URL http://www.R-project.org. 2010.

[54] Jones MH, Seeb JE, Warheit KI, Seamons TR, Quinn TP, Seeb LW. Consequences of emergence timing for the growth and relative survival of Steelhead Trout fry from naturally spawning wild and hatchery parents. Transactions of the American Fisheries Society. 2015; 144: 977–989. 10.1080/00028487.2015.1057346

[55] McGlauflin MT, Smith MJ, Wang JT, Young SF, Chen N, Lee YC, et al High-Resolution Melting Analysis for the Discovery of Novel Single-Nucleotide Polymorphisms in Rainbow and Cutthroat Trout for Species Identification. Transactions of the American Fisheries Society. 2010;139(3):676–84. 10.1577/t09-103.1 WOS:000277639200005.

[56] Smith MJ, Pascal CE, Grauvogel Z, Habicht C, Seeb JE, Seeb LW. Multiplex preamplification PCR and microsatellite validation enables accurate single nucleotide polymorphism genotyping of historical fish scales. Molecular Ecology Resources. 2011;11:268–77. 10.1111/j.1755-0998.2010.02965.x WOS:000287485100023. 21429180

[57] Foll M, Gaggiotti O. A Genome-Scan Method to Identify Selected Loci Appropriate for Both Dominant and Codominant Markers: A Bayesian Perspective. Genetics. 2008;180(2):977–93. 10.1534/genetics.108.092221 ISI:000260284400023. 18780740 PMC2567396

[58] Rousset F. GENEPOP ' 007: a complete re-implementation of the GENEPOP software for Windows and Linux. Molecular Ecology Resources. 2008;8(1):103–6. 10.1111/j.1471-8286.2007.01931.x ISI:000253827100016. 21585727

[59] Goudet J. FSTAT (Version 1.2): A computer program to calculate F-statistics. Journal of Heredity. 1995;86(6):485–6. ISI:A1995TL74700013.

[60] Weir BS, Cockerham CC. Estimating F-statistics for the analysis of population structure. Evolution. 1984;38(6):1358–70. ISI:A1984TY40400017. 28563791 10.1111/j.1558-5646.1984.tb05657.x

[61] Ryman N. CHIFISH: a computer program testing for genetic heterogeneity at multiple loci using chi-square and Fisher's exact test. Molecular Ecology Notes. 2006;6(1):285–7. 10.1111/j.1471-8286.2005.01146.x ISI:000235725600088.

[62] Excoffier L, Smouse PE, Quattro JM. Analysis of molecular variance inferred from metric distances among DNA haplotypes—application to human mitochondrial-DNA restriction data. Genetics. 1992;131(2):479–91. ISI:A1992HW75900021. 1644282 10.1093/genetics/131.2.479 PMC1205020

[63] Falush D, Stephens M, Pritchard JK. Inference of population structure using multilocus genotype data: Linked loci and correlated allele frequencies. Genetics. 2003;164(4):1567–87. ISI:000185248000029. 12930761 10.1093/genetics/164.4.1567 PMC1462648

[64] Pritchard JK, Stephens M, Donnelly P. Inference of population structure using multilocus genotype data. Genetics. 2000;155(2):945–59. ISI:000087475100039. 10835412 10.1093/genetics/155.2.945 PMC1461096

[65] Evanno G, Regnaut S, Goudet J. Detecting the number of clusters of individuals using the software STRUCTURE: a simulation study. Molecular Ecology. 2005;14(8):2611–20. 10.1111/j.1365-294X.2005.02553.x ISI:000229961500029. 15969739

[66] Earl D, vonHoldt B. STRUCTURE HARVESTER: a website and program for visualizing STRUCTURE output and implementing the Evanno method. Conservation Genetics Resources. 2012;4(2):359–61. 10.1007/s12686-011-9548-7

[67] Jakobsson M, Rosenberg NA. CLUMPP: a cluster matching and permutation program for dealing with label switching and multimodality in analysis of population structure. Bioinformatics. 2007;23(14):1801–6. 10.1093/bioinformatics/btm233 ISI:000249248300012. 17485429

[68] Waples RS, England PR. Estimating Contemporary Effective Population Size on the Basis of Linkage Disequilibrium in the Face of Migration. Genetics. 2011;189(2):633–44. 10.1534/genetics.111.132233 WOS:000296158500018. 21840864 PMC3189803

[69] Ostberg C, Hauser L, Pritchard V, Garza J, Naish K. Chromosome rearrangements, recombination suppression, and limited segregation distortion in hybrids between Yellowstone cutthroat trout (Oncorhynchus clarkii bouvieri) and rainbow trout (O. mykiss). BMC Genomics. 2013;14(1):570 10.1186/1471-2164-14-570 23968234 PMC3765842

[70] Berthelot C, Brunet F, Chalopin D, Juanchich A, Bernard M, Noël B, et al The rainbow trout genome provides novel insights into evolution after whole-genome duplication in vertebrates. Nat Commun. 2014;5 10.1038/ncomms4657 PMC4071752 24755649

[71] Gomez-Uchida D, Seeb JE, Smith MJ, Habicht C, Quinn TP, Seeb LW. Single nucleotide polymorphisms unravel hierarchical divergence and signatures of selection among Alaskan sockeye salmon (Oncorhynchus nerka) populations. BMC Evolutionary Biology. 2011;11 48 10.1186/1471-2148-11-48 ISI:000289411800002. 21332997 PMC3049142

[72] Hendry AP, Castric V, Kinnison MT, Quinn TP. The Evolution of Philopatry and Dispersal: Homing vs. Straying in Salmonids In: Hendry AP, Stearns SC, editors. Evolution Illuminated: Salmon and Their Relatives. New York: Oxford University Press; 2004 p. 52–91.

[73] Quinn TP. The Behavior and Ecology of Pacific Salmon and Trout. Seattle: University of Washington Press; 2005.

[74] Seeb LW, Habicht C, Templin WD, Tarbox KE, Davis RZ, Brannian LK, et al Genetic diversity of sockeye salmon of Cook Inlet, Alaska, and its application to management of populations affected by the Exxon Valdez oil spill. Transactions of the American Fisheries Society. 2000;129(6):1223–49. ISI:000167141400002.

[75] Palstra FP, Ruzzante DE. A temporal perspective on population structure and gene flow in Atlantic salmon (Salmo salar) in Newfoundland, Canada. Canadian Journal of Fisheries and Aquatic Sciences. 2010;67(2):225–42. 10.1139/f09-176 ISI:000274352400002.

[76] Ozerov MY, Veselov AE, Lumme J, Primmer CR. Temporal variation of genetic composition in Atlantic salmon populations from the Western White Sea Basin: influence of anthropogenic factors? BMC Genetics. 2013;14 10.1186/1471-2156-14-88 WOS:000326396200001. PMC3852729 24053319

[77] Hansen MM, Ruzzante DE, Nielsen EE, Bekkevold D, Mensberg KLD. Long-term effective population sizes, temporal stability of genetic composition and potential for local adaptation in anadromous brown trout (Salmo trutta) populations. Molecular Ecology. 2002;11(12):2523–35. WOS:000179492800007. 12453237 10.1046/j.1365-294x.2002.01634.x

[78] Waples RS, Yokota M. Temporal estimates of effective population size in species with overlapping generations. Genetics. 2007;175(1):219–33. ISI:000244180300020. 17110487 10.1534/genetics.106.065300 PMC1775005

[79] Thorstad EB, Fleming IA, McGinnity P, Soto D, Wennevik V, Whoriskey FG. Incidence and impacts of escaped farmed Atlantic salmon *Salmo salar* in nature. 2008.

[80] Schroder V, de Leaniz CG. Discrimination between farmed and free-living invasive salmonids in Chilean Patagonia using stable isotope analysis. Biological Invasions. 2011;13(1):203–13. 10.1007/s10530-010-9802-z ISI:000285359300022.

[81] Glover KA. Forensic identification of fish farm escapees: the Norwegian experience. Aquaculture Environment Interactions. 2010;1(1):1–10. 10.3354/Aei00002 ISI:000292392600002.

[82] Luikart G, Cornuet JM. Empirical evaluation of a test for identifying recently bottlenecked populations from allele frequency data. Conservation Biology. 1998;12(1):228–37. ISI:000072094300025.

[83] Leberg PL. Effects of Population Bottlenecks on Genetic Diversity as Measured by Allozyme Electrophoresis. Evolution. 1992;46(2):477–94. 10.2307/2409866 ISI:A1992HQ63100016. 28564024

[84] Roman J, Darling JA. Paradox lost: genetic diversity and the success of aquatic invasions. Trends in Ecology & Evolution. 2007;22(9):454–64. 17673331 10.1016/j.tree.2007.07.002

[85] Allendorf FW, Lundquist LL. Introduction: Population biology, evolution, and control of invasive species. Conservation Biology. 2003;17(1):24–30. 10.1046/j.1523-1739.2003.02365.x WOS:000180846200007.

[86] Frankham R. Challenges and opportunities of genetic approaches to biological conservation. Biological Conservation. 2010;143(9):1919–27. 10.1016/j.biocon.2010.05.011 WOS:000281125400001.

[87] BBC_News. Calbuco volcano erupts in Chile sending smoke and ash into the sky. 23 April 2015. Available http://www.bbc.com/news/world-latin-america-32425767. 2015.

