# Supplementary figures and images for: Temporal Genetic Variance and Propagule-Driven Genetic Structure Characterize Naturalized Rainbow Trout (Oncorhynchus mykiss) from a Patagonian Lake Impacted by Trout Farming
Source: PLoS One. 2015 Nov 6;10(11):e0142040. doi: 10.1371/journal.pone.0142040 (PMC4636326; doi:10.1371/journal.pone.0142040)

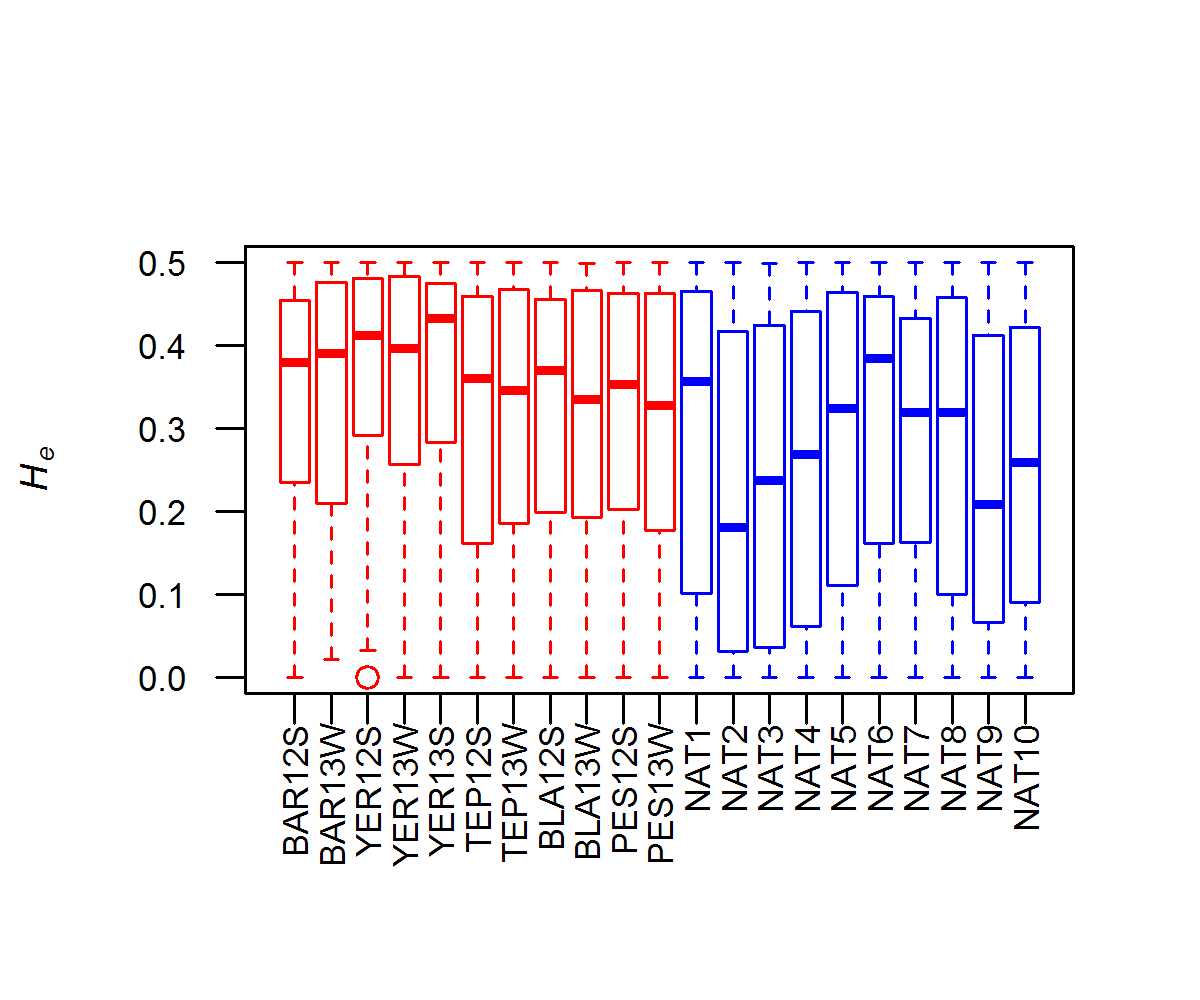

Supplement: S1 Fig — (TIF) [file pone.0142040.s001.tif]
